# Dynamic anthropomorphic thorax phantom for quality assurance of motion management in radiotherapy

**DOI:** 10.1016/j.phro.2024.100587

**Published:** 2024-05-06

**Authors:** Sara Abdollahi, Ali Asghar Mowlavi, Mohammad Hadi Hadizadeh Yazdi, Sofie Ceberg, Marianne Camille Aznar, Fatemeh Varshoee Tabrizi, Roham Salek, Matthias Guckenberger, Stephanie Tanadini-Lang

**Affiliations:** a Department of Physics, Faculty of Science, Ferdowsi University of Mashhad, Mashhad, Iran; b Department of Radiation Oncology, University Hospital Zurich, 8091 Zurich, Switzerland; c Department of Physics, Hakim Sabzevari University, Sabzevar, Iran; d Department of Medical Radiation Physics, Lund University, Lund, Sweden; e Division of Cancer Sciences, Faculty of Biology, Medicine and Health, University of Manchester, Manchester, United Kingdom; f Department of Radiation Oncology, Reza Radiotherapy and Oncology Center, Mashhad, Iran; g Department of Radiation Oncology, Mashhad University of Medical Science, Mashhad, Iran; h Département de physique, Université de Montréal, Montréal, Québec, Canada

**Keywords:** Lung SBRT, Dynamic anthropomorphic phantom, End-to-end test

## Abstract

**Background and purpose:**

Motion management techniques are important to spare the healthy tissue adequately. However, they are complex and need dedicated quality assurance. The aim of this study was to create a dynamic phantom designed for quality assurance and to replicate a patient’s size, anatomy, and tissue density.

**Materials and methods:**

A computed tomography (CT) scan of a cancer patient was used to create molds for the lungs, heart, ribs, and vertebral column via additive manufacturing. A pump system and software were developed to simulate respiratory dynamics. The extent of respiratory motion was quantified using a 4DCT scan. End-to-end tests were conducted to evaluate two motion management techniques for lung stereotactic body radiotherapy (SBRT).

**Results:**

The chest wall moved between 4 mm and 13 mm anteriorly and 2 mm to 7 mm laterally during the breathing. The diaphragm exhibited superior-inferior movement ranging from 5 mm to 16 mm in the left lung and 10 mm to 36 mm in the right lung. The left lung tumor displaced ± 7 mm superior-inferiorly and anterior-posteriorly. The CT numbers were for lung: −716 ± 108 HU (phantom) and −713 ± 70 HU (patient); bone: 460 ± 20 HU (phantom) and 458 ± 206 HU (patient); soft tissue: 92 ± 9 HU (phantom) and 60 ± 25 HU (patient). The end-to-end testing showed an excellent agreement between the measured and the calculated dose for ion chamber and film dosimetry.

**Conclusions:**

The phantom is recommended for quality assurance, evaluating the institution’s specific planning and motion management strategies either through end-to-end testing or as an external audit phantom.

## Highlights

• A valuable and robust phantom for comprehensive validation of the entire lung stereotactic body radiation therapy workflow.
• Accurate emulation of a patient’s size, anatomy, and tissue density.
• A phantom with key characteristics of thoracic breathing patterns.
• A valuable tool for imaging quality assurance of four-dimensional computed tomography and image guided radiotherapy.
• Suitable for dose measurements in inhomogeneous media and dose measurements in a moving tumor.

## 1 Introduction

Radiotherapy for moving targets presents a significant challenge and requires the implementation of motion management techniques to reduce uncertainties. These techniques must be rigorously validated via internal end-to-end tests or independent dosimetry audits using a realistic patient-imitating geometry [1]. Dynamic phantoms are frequently employed to perform end-to-end testing in radiotherapy motion management. There are different commercially available dynamic phantoms for simulating breathing patterns and ensuring the measurement quality of radiotherapy procedures. These phantoms often feature solid thorax geometries that can mimic translations and rotations for simple geometric targets. Their highlighted features include compactness and user-friendly platform-based designs, contributing to their ease of use. However, they often have a simplified geometry and do not emulate real patients, making it challenging to evaluate the performance of imaging systems in actual clinical scenarios.

Researchers have been compelled to create custom phantoms to address the limitations of commercial offerings for various quality assurance needs, spanning from dosimetry to imaging applications. The development of anthropomorphic phantoms aims to replicate a patient's anatomical characteristics, and the attenuation of imaging and radiation doses in human tissues due to the photoelectric and Compton effects, respectively. Key factors to consider in this context include physical density and electron density, both approximated from CT numbers typically expressed in Hounsfield units (HU) in CT imaging. As a review on anthropomorphic designs in research phantoms, Steidl *et al*. introduced a more sophisticated breathing phantom featuring a deformable thorax comprising film and ion chamber housing for dose measurement [2]. Although their phantom incorporated a commercial artificial skeleton, it used air to fill the lungs, which did not accurately replicate human tissue characteristics. Haas *et al*. developed a thorax phantom with a bone structure crafted from a blend of calcium carbonate and epoxy resin [3]. In their design, the lungs remained static, and the volume between the ribs and the static lungs changed in response to rib movement. Perrin *et al*. developed an anthropomorphic phantom to simulate thoracic motions, with soft tissue and bone closely resembling human tissues [4]. However, a limitation of their approach was the relatively low density of the foam material used for the lung.

The aim of this study was to develop a dynamic anthropomorphic phantom intended to enhance current models by improving the replication of human lung motion and tissue characteristics, specifically tailored for precise dosimetry in radiotherapy scenarios involving mobile lung tumors. The phantom is suggested for quality assurance purposes, serving to assess the institution's specific planning and motion management strategy in an end-to-end manner or as an external audit phantom.

## 2 Materials and Methods

### 2.1 Manufacturing of the phantom

**Fig. 1:**
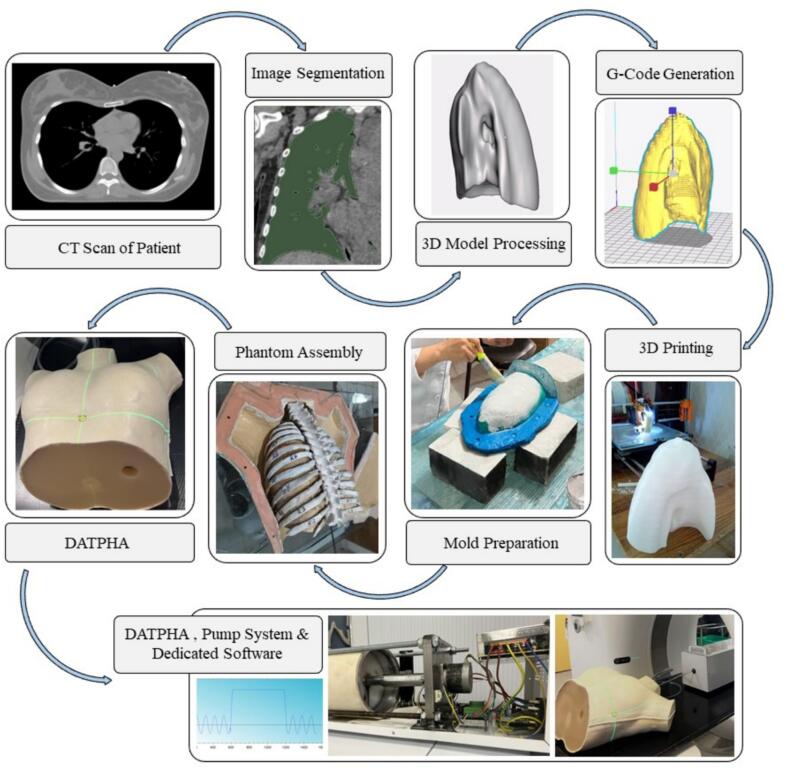
Flowchart outlining the procedural steps from the initial CT scan of the patient (top left) to the generation of phantom breathing facilitated by a custom-designed pump system and dedicated software.

We employed the Prusa i3 MK3 3D printer (Prusa Research, Czech Republic), which utilizes Fused Deposition Modeling (FDM) technology, to fabricate the phantom. A breast cancer patient's computed tomography (CT) scan served as a basis for generating casting molds using additive manufacturing for the body, lungs, heart, ribs, and vertebral column. The selection of this particular patient was based on her representative average anatomy, characterized by a body mass index of 23 and normal breast size. The CT scan was acquired at 120 kVp and had a 2 mm slice thickness. Fig. 1 illustrates a summarized depiction of the process via a flowchart, resulting in the construction of the phantom alongside a custom-designed pump system and a dedicated software for lung inflation and deflation. A comprehensive description of the manufacturing process was outlined within the Supplementary Materials A.

A Siemens Somatom Edge Plus CT scanner (Siemens Healthineers, Erlangen, Germany) was used to measure the CT number of the phantom components. The CT scan was acquired with a standard clinical imaging protocol (120 kV, 80 mAs, slice thickness 1 mm, medium-smooth Br38 kernel for reconstruction). ImageJ software [5] was used to analyze the CT number in the regions of interest (ROIs) for different tissues. ROIs were delineated in a circle shape at corresponding anatomical locations in lung, bone, and soft tissue in both patient and phantom images.

**Fig. 2:**
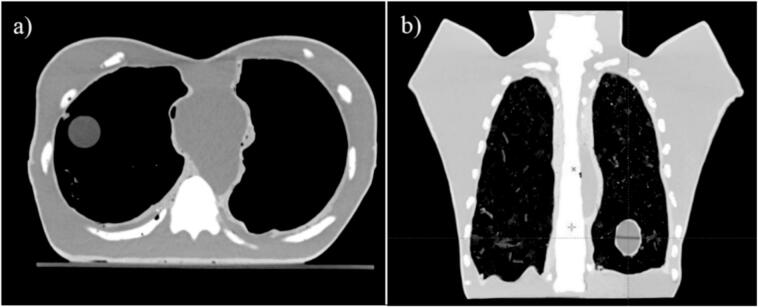
An axial CT slice with a water-filled sphere in the right lung (a) and a coronal CT slice with a movable tumor in the left lung (b).

A dynamic anthropomorphic thorax phantom (DATPHA) was successfully built. An axial CT slice of the phantom with a water-filled sphere in the right lung (a) and a coronal CT slice with a movable tumor in the left lung (b) were presented in Fig. 2. The dimensions of the phantom were 340 mm in length and 180 mm in height along the chest's midline, with a width of 310 mm at the body's mid-separation.

### 2.2 Motion control and dynamic tests

To simulate respiratory dynamics, a custom-made pump system and dedicated software were developed to cyclically inflate and deflate the lungs. This dynamic process induced synchronized movements in the chest and diaphragm while incorporating three-dimensional, non-isotropic tumor motion. Our approach involved utilizing the maximum air volume available within the capacity of our pumping system to fill the phantom, followed by assessing its impact on organ motion. Further elaboration on the software and hardware of the motion control system is available in the Supplementary Materials B.

The 4DCT scans of the phantom were acquired synchronously with the respiratory signal recorded by the Varian respiratory gating for scanners (Varian Medical Systems, Inc., Palo Alto, CA) and provided multiple 3D CT image data sets, sorted by 10 respiratory phases. The Real-time Position Management (RPM) block was positioned on the phantom's chest close to the xiphoid, as indicated by the intersection of lasers on the phantom in Fig. 1. When the phantom was run with a sinusoidal breathing pattern, this imaging tracked the motion of the tumor and skin surface to assess the external-to-internal motion correlation.

To quantitatively assess the extent of respiratory motion, a rigid image registration approach was used between the lowest exhalation (100 %) and highest inhalation (50 %) phases of the 4DCT dataset. To evaluate the reliability and uniformity of the phantom's motion patterns, a series of five 4DCT scans were performed over three months. These scans were intended to assess the degree of tumor positional variability during both deep inhalation and exhalation. To achieve this goal, the positions of the tumor were analyzed by registering CT images taken at corresponding respiratory phases based on bony anatomy landmarks.

### 2.3 End-to-end test

The end-to-end test followed a typical lung cancer patient workflow from CT simulation to treatment delivery. These tests encompassed two different dose delivery techniques and two different target sizes. Specifics regarding the techniques, organ delineation, and target definition and treatment planning and dose delivery analysis are presented in the Supplementary Materials C.

To evaluate the reproducibility of the film positioning inside the tumor, the phantom was placed on the treatment couch without any respiration. CBCT was utilized to align the phantom accurately for treatment, followed by delivery of a lung SBRT plan to the tumor. After removing the tumor, a new film was inserted, and the tumor was repositioned within the left lung for another round of treatment. This process was repeated three times. Irradiated films were then scanned together in two different setups as shown in Fig. S.4 in the Supplementary Materials C. In the image on the left, the dashed blue lines indicate where the film is placed within the tumor, which is represented by an orange shape combining a square and a half circle. The FilmQAPro software was utilized to extract lateral (x) and longitudinal (y) profiles, enabling analysis of the film's consistent positioning in both lateral and longitudinal directions.

## 3 Results

### 3.1 Manufacturing of the phantom

This phantom closely mimics the anatomical features of a human torso, including dimensions, shape, and tissue properties. The findings from the evaluation of CT numbers and tissue densities for various tissues have been condensed into Table 1.

**Table 1:** Material properties of the components of the phantom in comparison with patient. Lung properties observed during deep inspiration breath-hold (DIBH) and free-breathing (FB) states were presented. The mass densities were derived from the CT conversion curve. SD represents the standard deviation.

Organ	Phantom		Patient	
	HU ± SD	Mass density (g/cm^3^)	HU ± SD	Mass density (g/cm3)
Lung (DIBH)	−759 ± 103	0.2	−768 ± 130	0.23
Lung (FB)	−716 ± 108	0.31	−713 ± 70	0.3
Bones	460 ± 20	1.3	458 ± 206	1.16–––1.45
Heart	95 ± 8	1.05	50 ± 7	1.026
Soft Tissue	92 ± 9	1.05	60 ± 25	1.026
Movable Tumor	−70 ± 7	0.97		
Water-filled Sphere	−8 ± 9	1		

### 3.2 Motion control and dynamic tests

The image registration between deep inhalation and deep exhalation revealed a pattern similar to human respiration, where the inferior portion of the chest wall exhibits a greater anterior movement compared to the superior region. Likewise, the anterior part of the body exhibited greater lateral movement compared to the posterior section, mirroring typical human breathing dynamics. The maximum range of motion for the chest wall observed between the end of exhalation and the end of inhalation was between 4 mm (superior part) to 13 mm (inferior part) in the anterior direction and 2 mm (posterior part) to 7 mm (anterior part) in the lateral direction. The diaphragm motion in the superior-inferior (SI) direction was between 5 mm (medial part) to 16 mm (lateral part) for the left lung and 10 mm (medial part) to 36 mm (lateral part) for the right lung. The left lung tumor lesion displaced 14 mm ± 1 mm in the superior-inferior and anterior-posterior directions. Fig. S.2 in the Supplementary materials displays a fusion overlay of the phantom's deep inhale and exhale in both axial and sagittal planes, along with a zoomed-in view of the tumor's position in the sagittal plane. The reproducibility of the tumor position (with respect to the bony anatomy) in different respiratory phases over time (5 4DCT acquired over three months) was within 1 mm in the SI direction and <0.5 mm in all other directions.

### 3.3 End-to-end test

The dose differences between the calculated mean dose in the microDiamond sensitive volume and the measured dose by the detector for the large volume target were 0.2 % for the ITV plan, and 1 % for the gated plan. The dose differences for the small target were 1 % for the ITV plan and 3.4 % for the gated plan.

The gamma pass rates for both global and local normalization with different gamma criteria were shown in Table 2.

**Table 2:** The gamma pass rate results for film dosimetry of different dose delivery techniques for the small target.

	Global Gamma Pass Rate (%)			Local Gamma Pass Rate (%)		
	3 %,2 mm	5 %,1 mm	3 %,1 mm	3 %,2 mm	5 %,1 mm	3 %,1 mm
ITV plan	100	99.1	98.2	99.9	98.1	97.2
Gated plan	100	100	100	100	99.8	98.5

**Fig. 3:**
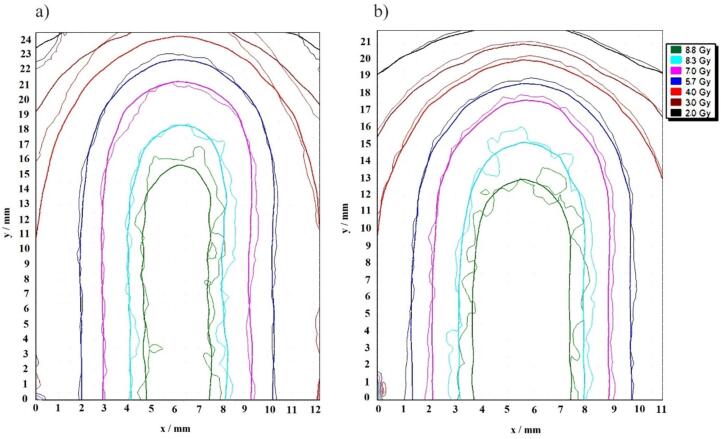
Isodose overlay comparison between the calculated and measured dose distributions for the ITV plan (left) and Gated plan for the phases of 10% to 90% (right). Thick lines illustrate the isodoses generated by the TPS, while thin lines show the measured dose measured by EBT3 films. The dark blue isodose lines specifically represent levels that align with 95% of the prescribed dose, meeting the necessary dosage for PTV coverage.

As shown in Fig. 3, the convergence of the 95 % calculated and measured dose (the dark blue isodose lines) for both dynamic delivery techniques confirmed the preservation of target coverage.

**Fig. 4:**
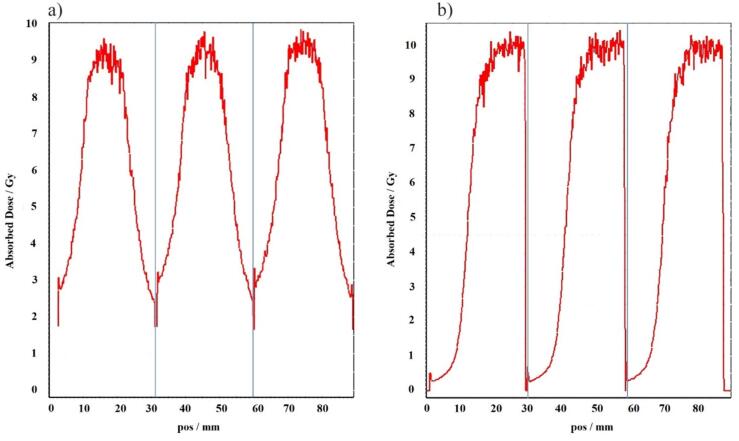
The lateral (a) and longitudinal (b) dose profiles extracted from irradiated films across three repetitions (separated from each other with blue lines) of a singular lung SBRT planned dose delivery. Both the maximum dose and dose gradient demonstrate notable similarities across the profiles. This consistent data highlights the reproducibility of film positioning within the tumor.

The reproducibility of film positioning within the tumor has been shown through lateral and longitudinal dose profiles extracted from scanned irradiated films using FilmQAPro software in Fig. 4. The profiles exhibit similarities in both maximum dose and dose gradient.

## 4 Discussion

This phantom closely replicates the anatomical characteristics of a human torso, including dimensions, shape, and tissue properties. As such, it serves as an advantageous tool for quality assurance (QA) throughout the radiotherapy treatment process. Its applications encompass QA of image-guided radiotherapy (IGRT), dose measurements in heterogeneous mediums, dose measurements in moving tumors, and end-to-end measurements.

The fabrication of the entire phantom via 3D printing facilitates the creation of personalized and intricate designs. However, this method imposes limitations on material selection, thereby affecting the replication of human tissue properties such as density and CT number. Conversely, utilizing 3D printing for the production of the casting mold offers flexibility in material choice for constructing the phantom. By separating mold fabrication from phantom production, adjustments to the phantom's material properties became more straightforward.

While silicon does not inherently match the properties of soft tissue, different silicone types and mixes with a density of about 1.08 g/cm^3^ were used in the literature to mimic the relevant CT number for soft tissue in CT imaging [[6], [7], [8], [9], [10]]. This material choice, although not aligning perfectly with typical soft tissue parameters, stands out as the closest available option. Our study explored diverse combinations of industrial silicone and silicone oil, encompassing various percentages of each component. Through these varied mixtures, our aim was to achieve a density closer to soft tissue, specifically 1.05 g/cm^3^, and a CT number of 92 HU. This was a novel approach initiated by our team for soft tissue emulation. The blend we discovered, comprising industrial silicone and silicone oil, represents the most optimal combination, we could find. Although increasing the percentage of silicone oil compared to industrial silicone might reduce the CT number and compound density, there is a limit to this adjustment. Any further increase in oil content would lead to a very fluid composition, hindering the ability to maintain the desired body mass formation.

Several studies showed that calcium-filled polymers provide both, favorable energy dependence, and attenuation values to mimic bone tissues [[3], [10], [11], [12]]. We have built on this knowledge and selected a bone mixture with a density resembling that of human bone, typically falling within the range of 1.2 to 1.9 g/cm^3^ [[10], [13]]. The CT number of cortical bone in the rib ranges from 500 to 750 HU. However, we specifically opted for a density and CT number close to the lower end of this spectrum to capture a balanced representation of both dense and soft bone characteristics. The higher range of CT numbers observed in the patient's bone can be attributed to the presence of dense bone with CT numbers between 600 and 1000 HU, as well as soft bone regions with CT numbers between 150 and 200 HU, in the ribs and vertebral column.

McGarry *et al.* conducted a comprehensive review of various studies focused on tissue-mimicking materials employed in imaging and therapy phantoms [14]. Within their analysis, it was observed that polyurethane (PU) foam exhibited the capability to mimic lung characteristics, primarily owing to its density variability during compression. This particular attribute is notably advantageous for simulating the dynamic processes of inhalation and exhalation. Perin et al. utilized a low-density PU foam (density: 0.03, CT number: −980 HU) in their work, which, although not precisely mirroring human lung tissue, possessed an appropriate elastic modulus conducive to achieving realistic extension during lung inflation [4]. Shin *et al.* introduced iodine as a contrast agent to enhance the foam's density and make it closer to the human lung [15]. However, it should be noted that their lung phantom, while valuable for assessing imaging quality, is not suitable for radiation dosimetry.

The phantom, as featured in the present study, is the only dynamic phantom that has density and CT number similar to a real patient and is suitable for precise dosimetry in scenarios involving mobile lung tumors.

The lack of control over the extent of tumor motion in different directions may be viewed as a limitation of the current study. Studies have shown that the only method to achieve controlled motion in various directions within the phantom body or tumor is through the use of distinct actuators for each component. In pneumatic-driven phantoms when there is no specific actuator for the tumor motion, the extent of motion for the body or tumor in various directions cannot be precisely controlled, nor can the tumor be manipulated to move specific distances along the x, y, and z axes. However, the primary objective of this study was to develop an anthropomorphic dynamic phantom for end-to-end testing of lung SBRT treatment, prioritizing overall functionality over predetermined tumor motion. However, we have demonstrated that the motion is reproducible, suggesting QA can be accomplished by consistently using the same pressure settings and comparison to static positions (pumped with different pressure).

The extent of tumor motion in this phantom is slightly less compared to findings from other phantom studies, particularly those that used an independent motor to control tumor motion [[16], [17]]. However, this range is similar to tumor motion in patients [[18], [19], [20]]. Knybel et al analyzed 145 patients and reported a mean tumor motion amplitude of 6 ± 2.2 mm, with motion ranging from 0.8 to 18.7 mm in the SI direction. Notably, the tumor motion extend of 14 mm is similar to commonly employed in studies investigating motion-related aspects [[1], [19], [21]]. The limited range of movement in the left lung's diaphragm, as compared to the right lung, is primarily attributed to the presence of a sizable tumor attached to the left diaphragm. The similarity in length between the tumor and the canal was intentionally chosen to facilitate the insertion and removal of the tumor. The authors are actively exploring ways to address this constraint in the next revision of the phantom, probably by implementing a larger pump system and a more powerful motor, to increase the air volume within the lungs. For the next revision we also aim to explore technical strategies to eliminate the air bubbling observed around the peripheral regions and structural boundaries during the initial CT scan of the assembled phantom.

The waving appearance of the right diaphragm in the phantom is attributed to the absence of the liver beneath the right lung, a scenario that typically pushes the right diaphragm upward, creating a dome-shaped contour due to the liver's presence in actual patients. In our phantom, we replicated the lung mold from the patient's CT scan, omitting the liver structure. Consequently, the excess length of the diaphragm leads to a wavy formation in the absence of the liver's usual support.

This study showed that the phantom accurately mimics the anatomy of the human torso, including its size, shape, and tissue properties. It shows a closer resemblance in tissue density and CT number compared to other phantoms of its kind. Furthermore, when used for to test the accuracy of an SBRT treatment of lung cancer it showed high accuracy in dose delivery, as measured by film dosimetry. Therefore, the phantom proves to be a valuable and reliable tool for thoroughly assessing and validating the entire workflow of lung SBRT.

## CRediT authorship contribution statement

**Sara Abdollahi:** Conceptualization, Methodology, Validation, Formal analysis, Investigation, Writing – original draft, Visualization. **Ali Asghar Mowlavi:** Writing – review & editing, Supervision, Funding acquisition. **Mohammad Hadi Hadizadeh Yazdi:** Supervision, Funding acquisition, Writing – review & editing. **Sofie Ceberg:** Writing – review & editing. **Marianne Camille Aznar:** Validation, Writing – review & editing. **Fatemeh Varshoee Tabrizi:** Resources, Writing – review & editing. **Roham Salek:** Writing – review & editing. **Matthias Guckenberger:** Validation, Writing – review & editing. **Stephanie Tanadini-Lang:** Validation, Writing – review & editing, Supervision.

## Declaration of competing interest

The authors declare that they have no known competing financial interests or personal relationships that could have appeared to influence the work reported in this paper.
