# Supplementary material for: Dynamic anthropomorphic thorax phantom for quality assurance of motion management in radiotherapy
Source: Phys Imaging Radiat Oncol. 2024 May 6;30:100587. doi: 10.1016/j.phro.2024.100587 (PMC11137593; doi:10.1016/j.phro.2024.100587)
Supplement: Supplementary Data 2 [file mmc2.docx]

### **Supplementary Materials**

## Manufacturing of the phantom

The CT of the patient was imported into 3D Slicer (version 5.0.2), a free open-source software application for medical image computing, to segment the image into regions of interest and create 3D models of specific tissue types. 3D Slicer generated the Standard Tessellation Language (STL) data sets for segmented models based on the threshold method for bone, lung, and soft tissue differentiation. Standard thresholds were used for segmentation. The soft tissue model was enlarged by 5 mm to fabricate the body contour mold, and then the original model was subtracted from the enlarged one. Different data files with STL format generated for lung, vertebra column, ribs, heart, and body contour were then imported into Autodesk Meshmixer (version 3.5; Autodesk Inc., San Rafael, CA, USA) for further processing. Surface mesh inspection of the model was done for integrity, watertightness, and manifold. Spikes, holes, and unwanted connections between parts were then manually edited. The edited STL files were then converted to G-code using Ultimaker Cura 5.4 (Ultimaker B.V., Utrecht, Netherlands) and exported to the Prusa i3 MK3 3D printer.

The FDM printed output served as the initial pattern form for building the fiberglass plug. Polylactic acid (PLA) was selected as the printing material due to its widespread availability, ease of printing, and environmentally friendly characteristics. The initial stage of the fiberglass mold-building procedure involve d the creation of a plug, mirroring the exact dimensions of the object to be manufactured. For the production of molds encompassing the heart, lungs, ribs, and body, individual clay partitions were carefully constructed around each section of the 3D printed component. Several dimples were made on the partitions, serving as registration keys for the initial half of the mold. Subsequently, layers of reinforcement material were applied to fortify and safeguard the plug. In the composite manufacturing process, we employed the hand lay-up technique. To commence, the refined surface of the plug was coated with a release anti-adhesive agent, preventing unwanted adhesion of polymer materials. Following this, a layer of epoxy resin gel coat was applied to the plug's surface. This gel coat, serving as the initial resin layer in the mold, offered protection against chemical exposure, weathering, humidity, and environmental aggressors. To catalyze the curing reaction and enable room-temperature curing, a catalyst was introduced into the resin. Fillers were subsequently incorporated into the mixture to enhance viscosity and resistance. Once the gel coat had fully cured, the next phase involved saturating the plug with chopped glass fiber strands to reinforce the structure. After curing the outermost skin layer, additional layers of fiberglass were progressively added to fortify the mold further. Ultimately, the completed mold was carefully separated from the plug, and this process was replicated for the body, two lungs, ribs, and heart components.

Figure S.1 shows the molds prepared for the left lung with housing for the movable tumor, heart, ribs, and vertebral column. For vertebral column manufacturing, the 3D-printed vertebral column was used as a positive frame. A negative imprint of this model was obtained by immersing the 3D-printed column model in a silicon bath inside a mold box. The vertebral column was then removed from the mold after allowing a full cure for silicon. The mold box was then reassembled around the cut mold, and the bone-mimicking material was then poured into the mold. To create the lungs, an initial 2mm-thick shell layer was formed within the mold and later removed once it had dried. A cavity was incorporated into the left lung diaphragm to allow the placement of a tumor.


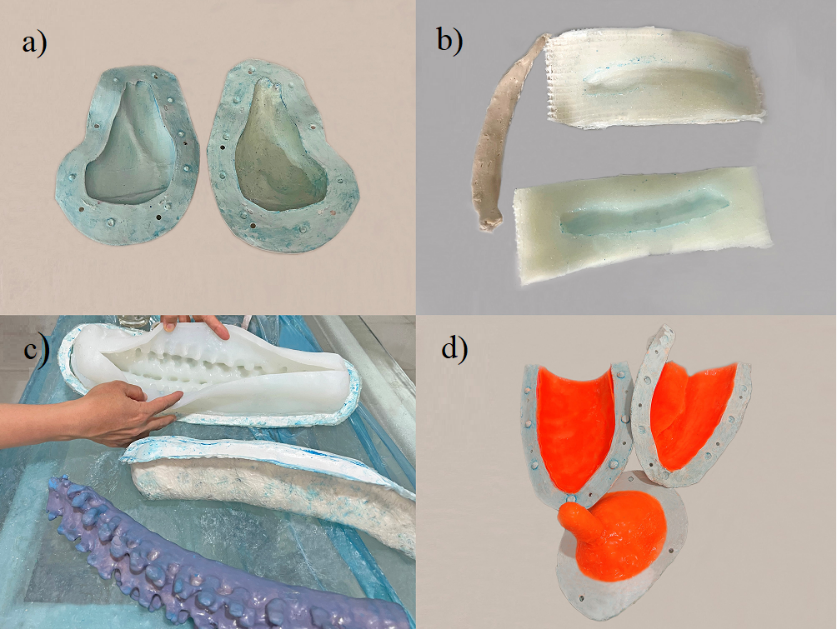


Figure S.1. The molds for the heart (a), ribs (b), vertebral column (c), and the left lung with housing for the movable tumor (d)

To replicate soft tissue properties, multiple combinations of industrial silicone and silicone oil were tested. Through this exploration, we identified the optimal ratio of four parts industrial silicone to five parts silicone oil, which was closer to the CT number of soft tissue. This method marks the first utilization of such an approach in this field of study. For bone tissue emulation, a composite mixture of polyester resin and calcium carbonate at a ratio of five to two was utilized. For simulating lung tissue, we utilized a composite formulation of polyurethane (PU) foam and barium sulfate, blended at a ratio of twenty-five parts PU foam to four parts barium sulfate. Barium sulfate served as an additive to match the CT number of the PU foam with that of actual lung tissue.

Three removable tumor structures were custom-designed using Polyurethane (density: 0.7 g/cm³) to integrate radiochromic films, a PTW 60019 microDiamond detector, or a PTW 30016 PinPoint 3D ion chamber (PTW, Freiburg, Germany), respectively. Each detector serves specific dosimetric purposes, and only one can be inserted into the phantom at a time. Figure S.2 depicts the tumor's cylindrical form, measuring 30 mm in diameter and 55 mm in height, along with its hemispherical top of equivalent diameter. This tumor perfectly occupies the 70 mm-long canal within the phantom. In the right lung, a water-filled sphere was introduced to replicate a very dense tumor. Subsequently, both lungs were filled with composite foam that had been processed into small pellets. Two separate tubes were inserted, one into each lung. These tubes, each measuring 10 mm in diameter, were securely affixed and sealed using silicon material. Additionally, a PVC tee was used to facilitate air infusion into each lung individually, with the tube outside the body measuring 500 mm in length and connected to the tee. The anthropomorphic phantom was eventually assembled, and the remaining space was filled with the soft tissue mimicking material.


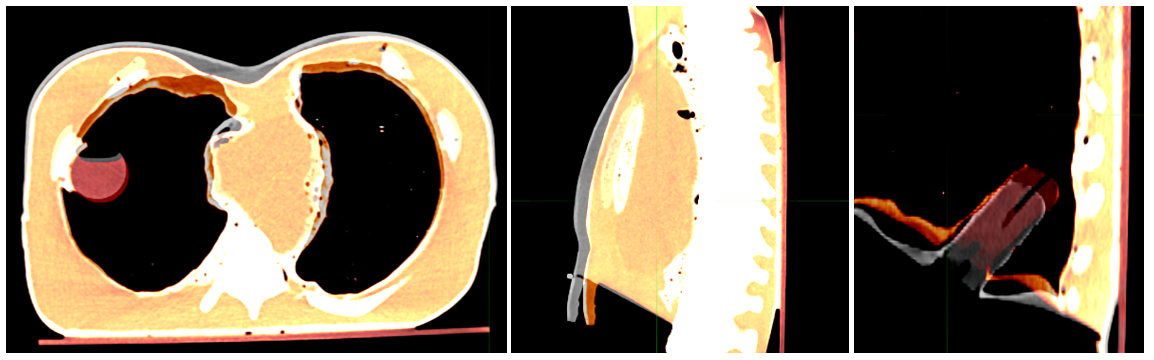
Figure S.2. Fusion overlay of deep inhale and deep exhale of the phantom; axial plane (left), sagittal plane (middle), zoomed-in view on the tumor’s position in sagittal plane (right).

1. Motion Control System

We developed a dedicated software to control the motion of the phantom. It offers users the flexibility to customize the airflow pumped into the system. This influences the generated respiratory waveform to suit specific requirements, allowing adjustments for amplitudes, frequencies, speeds, and offsets. Furthermore, it provides the functionality to import patient-specific breathing curves in either free-breathing or deep inspiration breath-hold modes, utilizing a comma-separated values (CSV) format for data representation. The software has an integrated breath hold function for convenient testing of gating protocols as needed. It can make adjustments to any captured or imported waveforms using different editing options and filters.
A four-axis computer numerical control (CNC) controller board was used to create the necessary commands for the required movements and desired speed. Additionally, a dedicated driver board was utilized to execute the controller commands and generate electrical pulses to initiate the stepper motor. This stepper motor serves as the primary driving force for the cylinder and piston, regulating air pressure within the system. A limiting switch further enhances operational precision, determining the initial and final limits of piston movement.

1. End-to-End Test

The 4DCTs of the phantom with tumor housing the film or microDiamond detector were imported into the Eclipse treatment planning system (Eclipse 16.0, Varian Medical Systems, Inc., Palo Alto, CA). The end-to-end tests were performed for two different dose delivery techniques and two different target sizes.

The movable structure, referred to as a tumor or large target, had the shape of a cylindrical structure with a height of 4.5 cm, featuring a hemisphere at one end with a diameter of 3 cm. Inside this large tumor structure, a smaller target was delineated, comprising a hemisphere with a diameter of 1.5 cm and a cylindrical portion measuring 3.5 cm in height. These two target structures of different sizes were employed as planning targets in lung SBRT plans.

The first technique was the ITV-based technique as the most widely accepted approach for treatment planning in lung stereotactic body radiation therapy (SBRT) [1,2]. In this passive strategy, the detected motion in 4DCT was delineated as an ITV. The tumor was delineated in all 10 phases of the 4DCT and the ITV was generated as the sum of these tumor structures. The second method was an active respiratory motion management technique as free-breathing gated in 10% to 90% of the breathing cycle in inhale. The GTV was delineated in the respiration phases of 10% to 90% in inhale. In both plans, PTVs were then created with 5mm isotropic expansion to the ITV and GTVs to account for any geometrical error or set-up uncertainty. The relevant critical structures, such as two lungs excluding the target and spinal canal, were delineated for dose reporting.

Two Rapid Arc plans using a 6MV flattening filter free (FFF) beam with four coplanar half arcs were optimized and calculated on the average intensity projection image set for the motion-encompassing and respiratory gated plans. The respiratory gate was set to 30% duty cycle in exhale resulting in a 3.5 mm gating window for external residual motion. The dose calculations were performed using the Acuros XB 16.6.04 algorithm (Eclipse 16.0, Varian Medical Systems, Inc., Palo Alto, CA) with a 1 mm calculation grid size. The prescription dose was 45 Gy in five fractions, with 100% of the prescribed dose to at least 95% of the planning target volumes (PTV), and all hot spots (139–152%) fell within the internal target volume (ITV) and gross tumor volume (GTV). Jaw tracking was activated during plan optimization to minimize out-of-field leakage. The following clinical goals were met in the SBRT plans: PTV V100% > 95%, Conformity Index ≥1, Conformity Index < 1.2; ITV V135% > 95%, D0.1cc < 156%, D0.1cc > 152%; Lung Dmean < 10 Gy; Spinal Cord expanded by 5 mm D0.1cc <13 Gy.

Figure S.3 displays the dose distribution of the lung SBRT treatment plan (motion-encompassing technique) in the sagittal and coronal views.


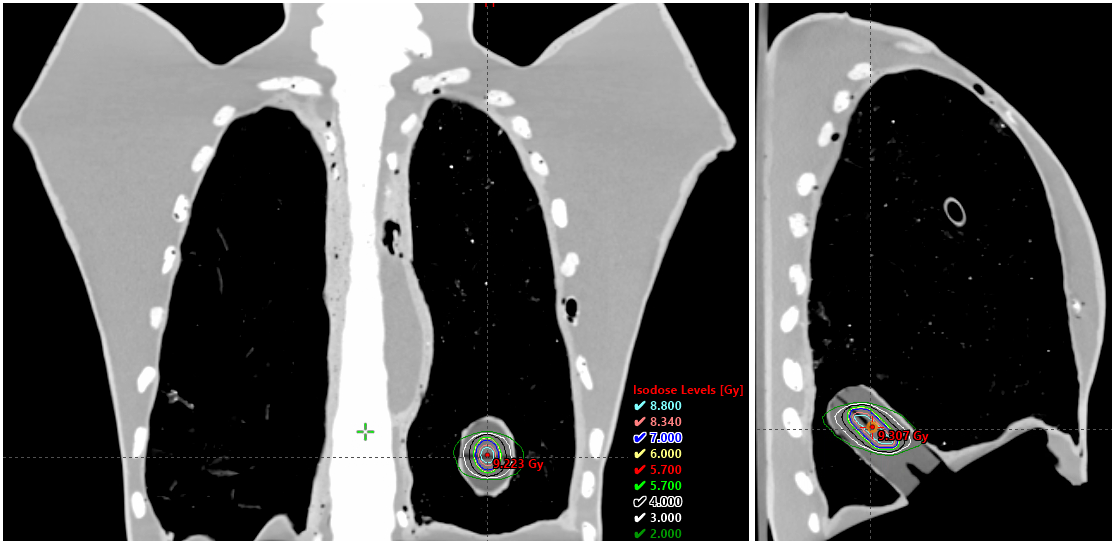


Figure S.3. Lung SBRT dose distribution; isodose overlay on axial slice of planning CT (left) isodose overlay on sagittal slice of planning CT (right)

The planar dose in the film plane was then exported to FilmQAPro software (Ashland ISP Advanced Materials, NJ) to compare with the measured dose. Treatment plans were then delivered for one fraction of 6 Gy to the DATPHA phantom containing different detectors using Varian Edge linac with a high-definition multi-leaf collimator. In ensuring accurate film dosimetry with EBT3 films, the fraction dose was adjusted from 9 Gy to 6 Gy before the actual delivery of the dose. This modification guaranteed that the recorded dose on the EBT3 films stayed below 10 Gy. As a result, this adjustment led to a maximum target dose ranging between 9 to 10 Gy in the treatment plan.

The point dose measurement was performed using a PTW microDiamond detector and PTW UNIDOS webline electrometer. The measured doses were then compared to the mean dose in the sensitive volume of the detector in each plan.

The 2D dose distribution was measured by the Gafchromic EBT3 film (Ashland ISP Advanced Materials, NJ) from a single batch in the coronal plane. For absolute calibration a film patch was irradiated using a 6 MV linear accelerator, with a traceable calibration to the Federal Institute of Metrology (METAS, Köniz, Switzerland) at the same time as the phantom film. The film was analyzed 24h after the irradiation of the films using FilmQA software. An Epson Expression 12000XL flat-bed scanner (Seiko Epson Corp, Nagano, Japan) was used to scan the calibration and plan quality assurance films in red–green–blue format with 150 dots per inch using transmission mode with no corrections. Films were placed in the center of the scanner bed to have better scanner response uniformity. A clear glass plate was placed over the films to reduce the dose uncertainty due to the Callier effect [3,4]. The isocenter of the film was aligned with the isocenter of the treatment planning system and dosimetric accuracy of different dose delivery techniques was assessed using the FilmQAPro software. The global and local gamma pass rates, employing 5% and 3% dose differences and 1mm distance-to-agreement criteria were evaluated for Gafchromic EBT3 film dosimetry. The planning data set was used as the reference and the dose cut-off thresholds were 10% of the maximum dose for both normalization methods. Gamma pass rates were solely reported for the small target, as irradiating the film with a plan intended for the large target leads to the loss of information regarding the penumbra. Consequently, we can only assess the target dose and not the dose gradient. While the delivered dose closely matched the planned dose for the large target, achieving a 100% pass rate for both 3% and 5% dose difference criteria, we chose not to report a gamma pass rate due to the absence of dose gradient information.


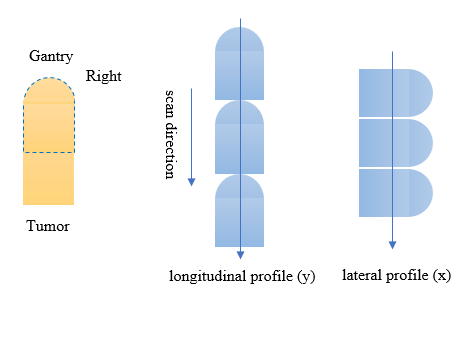


Figure S.4. Position (dashed blue line) and orientation of the film inside the tumor (left), lateral profile passing through three irradiated films (middle) in the first scan, longitudinal profile passing through three irradiated films (right) in the second scan

### **Supplementary References**

[1] Guckenberger M, Andratschke N, Dieckmann K, Hoogeman MS, Hoyer M, Hurkmans C, et al. ESTRO ACROP consensus guideline on implementation and practice of stereotactic body radiotherapy for peripherally located early-stage non-small cell lung cancer. Radiother Oncol. 2017;124:11-7. <https://doi.org/10.1016/j.radonc.2017.05.012>

[2] Ehrbar S, Jöhl A, Tartas A, Stark LS, Riesterer O, Klöck S, et al. ITV, mid-ventilation, gating or couch tracking–A comparison of respiratory motion-management techniques based on 4D dose calculations. Radiother Oncol. 2017;124:80-8. <https://doi.org/10.1016/j.radonc.2017.05.016>

[3] Niroomand‐Rad A, Chiu‐Tsao ST, Grams MP, Lewis DF, Soares CG, Van Battum LJ, et al. Report of AAPM task group 235 radiochromic film dosimetry: an update to TG‐55. Med Phys 2020;47:5986-6025. <https://doi.org/10.1002/mp.14497>

[4] Palmer AL, Bradley DA, Nisbet A. Evaluation and mitigation of potential errors in radiochromic film dosimetry due to film curvature at scanning. J Appl Clin Med Phys 2015;16:425-31. <https://doi.org/10.1120/jacmp.v16i2.5141>
